# Factors of Obesity and Metabolically Healthy Obesity in Asia

**DOI:** 10.3390/medicina58091271

**Published:** 2022-09-13

**Authors:** Bryan J. Mathis, Kiyoji Tanaka, Yuji Hiramatsu

**Affiliations:** 1 International Medical Center, University of Tsukuba Hospital, Tsukuba 305-8576, Ibaraki, Japan; 2 Faculty of Health and Sport Sciences, University of Tsukuba, Tsukuba 305-8576, Ibaraki, Japan

**Keywords:** body mass index, diabetes, metabolically healthy obesity, obesity

## Abstract

The East Asian region (China, Japan, and South Korea) is comprised of almost 1.5 billion people and recent industrialization has brought with it a pandemic of rising obesity, even in children. As these countries are rapidly aging and functioning at sub-replacement birthrates, the burgeoning costs of obesity-related care may threaten socialized healthcare systems and quality of life. However, a condition called metabolically healthy obesity (MHO) has been found to be without immediate cardiopulmonary or diabetic risk. Thus, maintenance of the MHO condition for the obese in East Asia could buffer the burden of long-term obesity care on medical systems and knowledge of the biochemical, genetic, and physiological milieu associated with it could also provide new targets for intervention. Diverse physiological, psychological, environmental, and social factors play a role in obesogenesis and the transition of MHO to a metabolically unhealthy obesity. This review will give a broad survey of the various causes of obesity and MHO, with special emphasis on the East Asian population and studies from that region.

## 1. Introduction

Obesity is a global health crisis that has reached East Asia (China, Japan, and South Korea) and its population of over 1.5 billion people. After industrialization, the availability of processed foods and Westernization of dietary trends have resulted in an estimated 40.9% overweight/obese prevalence among adults in the Asia–Pacific region, with an estimated economic impact of up to 12% of total health care spending on obesity or related diseases [1]. This will only worsen as the population ages.

Obesity is a chronic, progressive disease of caloric energy storage that manifests as excess visceral or subcutaneous lipid deposition. Obesity is defined as a body mass index (BMI) value of 30.0 kg/m^2^ (kg per m^2^ of body surface) or higher in Western countries or 25.0 kg/m^2^ or higher in Asian body types. Rather than the chief cause, current thought proposes that overeating is actually a manifestation of psychological, biochemical, genetic, or neurological imbalances and studies are currently attempting to delineate factors that increase obesity risk. A systemic condition, surplus adipose tissue generates inflammation through secretion of cytokines while the neuroendocrine and metabolic energy balance systems resist loss of stored fat as a conserved survival mechanism. Thus, obesity is not disease of storage, but a metabolic condition that resets energy homeostasis and inflicts gradual damage to the cardiopulmonary and glucose management systems. Overweight and even obese individuals within the first stages of the Edmonton Obesity Staging System (EOSS) are metabolically healthy, but the sustained biochemical burden of excess fat gives way to permanent glucose dysregulation (type 2 diabetes) which, in turn, greatly increases cardiovascular risk [2]. Obesity without this concomitantly increased cardiopulmonary risk is termed metabolically healthy obesity (MHO) while metabolically unhealthy obesity (MUHO) represents the endpoint of obesogenesis, but increases in cardiovascular, cancer, and all-cause mortality seen in numerous studies.

From the economic standpoint of the rapidly aging Asian population alone, an obesity pandemic would be unsustainable, as care for type 2 diabetes and cardiovascular diseases are expensive and long-term commitments. Additionally, obesogenesis in East Asian children is also increasing, which will affect population-level quality of life and further increase demand on limited resources for obesity-related care [3]. Therefore, if East Asians currently suffering from obesity can maintain MHO status for as long as possible before intervention, it would buffer the burden on the socialized medical systems found in East Asia as well as improving quality of life with regard to metabolic syndrome. However, since excessive fat has been found to increase peak loading of the musculoskeletal movement chain, the effect of weight stress could increase the failure rate of hip implants in the aging and cause balance issues even in younger populations [4,5] Thus, even if it not a state of total health, MHO is preferable to MUHO since a healthy metabolism may facilitate weight loss through exercise by reducing weight stress on the legs and hips.

In general, the first-line treatment strategy of “eat less, move more” is ineffective for MUHO in the long-term (partly due to obesity effects on the movement chain’s peak loading stress) but, as MHO is characterized by cardiopulmonary fitness and normal glucose regulation, maintenance of MHO and weight loss through diet and exercise may be possible in these individuals if targeted interventions remove known causes of MUHO pathogenesis [4]. While bariatric surgery (expensive and permanent) has shown some promise, obesity is a nearly incurable disease and, thus, prevention of obesogenesis and maintenance of MHO are of utmost importance to relieve the socialized medical systems of East Asia. However, in-depth analyses of the causes of obesity and potential factors for shifting from MHO to MUHO in East Asia are scarce.

Objective: This review summarizes causative factors for obesity and mentions differences between MHO and MUHO, especially with regard to biochemical, lifestyle, and social factors. The impact of MHO and MUHO in the unique milieu of East Asian populations from the standpoint of peer-reviewed clinical studies is also discussed.

## 2. Factors in Metabolically Healthy and Unhealthy Obesity

### 2.1. Age

Aging bodies tend to suffer increased risk of metabolic syndrome, with a Norwegian study of 10,206 adults finding 47.2% (male) and 64.4% (female) prevalences in the 80–89 age range [6]. This indicates that a large percentage of obese adults, even those with MHO, will eventually transition into MUHO status as they age and their resting metabolic rate and glucose control decrease, especially in post-menopausal women [7,8].

In East Asia: Asia is facing a demographic problem of aging, as exemplified in “elderly” societies such as China, which is currently experiencing growth rates in the elderly up to five times higher than total population growth [9]. Japan, where over 25% of the population is elderly and birth rates are below replacement, will also encounter infeasibly high societal costs for obesity-related disease similarly to South Korea with 14% of the total population aged 65 or over [10,11]. If even metabolically healthy obesity continues at the present rate, these new elderly individuals will eventually face the pathogenesis of diverse obesity-related diseases that will heavily strain the socialized healthcare systems common to East Asia.

### 2.2. CICO vs. CIM

The Calories In Calories Out (CICO) model, or energy balance model (EBM), treats every calorie as equal without accounting for the variability of resting energy expenditure based on time of day or other factors. While over 60–70% of daily caloric expenditure stems from basal metabolism, around 10% comes from the thermogenic effect of food intake (the energy used for digestion and nutrient assimilation) while the remaining 20% or so is the energy expended to move or exercise [12]. In this model, all calories are equal from an obesogenic perspective and substitutions (e.g., replacing fat calories with carbohydrate calories) are expected to have an effect dictated by simple mathematics (if calories in < calories out, then weight loss will occur). However, this model does assert that the brain tightly controls energy balance within the body through a network of nervous/endocrine messaging and that modern, processed food is confusing the neurological system with regard to food reward and dopamine release [13]. In this regard, the EBM is considered as a holistic system that measures caloric demand by internal body sensors but does not control for the degradation of assimilative and communicative machinery within the body (e.g., the inability of brains affected by leptin resistance to respond properly to leptin signaling [13].

The Carbohydrate–Insulin Model (CIM), on the other hand, focuses on the differential effects of metabolic glucose dysregulation and hormonal action on shifting calories from even healthy foods directly into fat via sustained increases in insulin response after meals [14]. In this model, processed carbohydrates (especially fructose and other insulinogenic starches) and excess protein spike insulin as a defensive response against uncontrolled blood sugar, driving the liver to convert sugars directly into heart- and artery-damaging lipids (blood triglycerides, cholesterol, and visceral fat) [14]. This effect is mirrored in type 2 diabetics, who receive insulin shots and experience a concomitant and tightly associated weight gain [15]. This theory is also bolstered by studies in non-diabetic obese patients who take metformin (gluconeogenesis inhibitor) or rosiglitazone (insulin response enhancer) and lose significantly more body weight than controls [16,17,18]. This model, however, does not take into account non-carbohydrate obesogenesis and insulin control, which could be substantial in individuals who consume excessive fat on low-carbohydrate diets and continue to gain non-muscle body weight [13].

In East Asia: The metabolic model may be relevant to prevent the sequelae of MUHO in East Asia as a health insurance study in Japan that reviewed 2548 total patients of normal or obese statuses taking either metformin or dipeptidyl peptidase-4 (glucose metabolism) inhibitors found that metformin resulted in a significant HbA1c reduction, increasing insulin sensitivity independent of BMI [19]. However, a study of 53,469 Singaporean adults of Han Chinese heritage found that, while the carbohydrate load of the typical Asian diet was not related to heart disease pathogenesis, transitioning from simple carbohydrates to produce and slow-digesting whole grains did reduce risk significantly [20]. Taken together, study results indicate that the CICO model could be outdated and MUHO is most likely driven, at least in part, by insulin resistance. This could explain why a majority of MUHO cases proceed to type 2 diabetes while pathogenesis in MHO is delayed significantly because these individuals still retain a normal glucose response and insulin sensitivity. Thus, with regard to MHO, shifting Asians from white rice to whole grains and produce could be protective against MHO to MUHO progression.

### 2.3. Damage from Reactive Oxygen Species

Metabolic damage results from excessive reactive oxygen species (ROS) generated by chronic inflammation, NADPH oxidases during hyperglycemia, polyol and hexosamine sugar shunting, excess blood lipids that generate superoxide anion radicals, and increased hydroxyl radicals generated by sustained, high leptin levels that also generate nitrogen radicals [21,22]. Over time, this cumulative damage could induce a shift from MHO to MUHO due to excess NADH that imbalances the membrane proton gradient and reduces insulin sensitivity through a vicious cycle mediated by JNK, p38MAPK, and NF-*κ*B that phosphorylate IRS-1 and IRS-2 insulin response proteins [21,23,24,25]. While Nrf2, a master antioxidant transcription factor, would normally be activated by enhanced ROS production, excess activation of NF-*κ*B may downregulate or blunt the endogenous Nrf2 response by upregulating Nrf2 constitutive repressor Keap1 via p65 under non-autophagic conditions (excessive glucose) [24]. Excessive processed carbohydrate consumption in the East, typically from white rice and white flour or instant noodles, may thus be causative in driving the pathogenesis of ROS-induced damage through chronic upregulation of Nrf2-repressing factors under hyperglycemic conditions.

**Figure 1:**
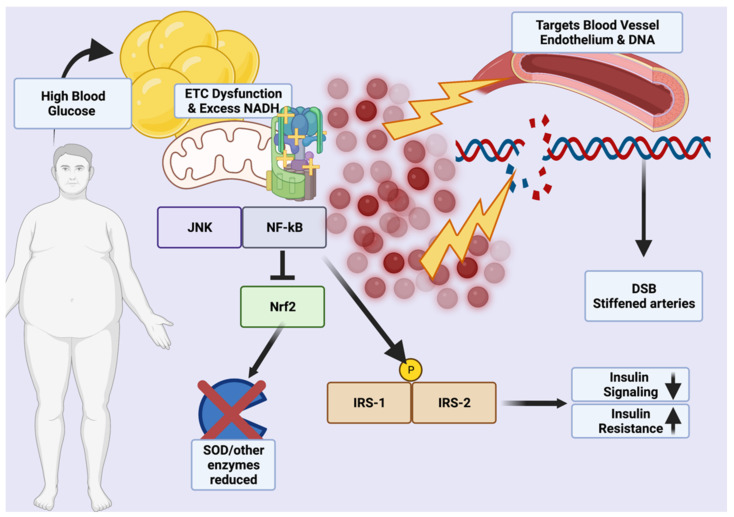
**Sources and targets of reactive oxygen species damage in obesity.** A multifactorial generation of reactive oxygen species comes from blood glucose dysregulation, reductions in detoxifying enzymes, and the adipocytes themselves. Targets include double-stranded DNA and the endothelial lining of blood vessels [21,23,24,25,29,32,33]. DSB = double-strand break Created in BioRender.com.

Of particular importance is the effect of ROS on the white and brown adipose tissue (WAT/BAT), the balance of which is dysregulated in the obese due to impairment by adipocyte hypertrophy caused from excessive energy buffering [26]. Obese individuals are known to carry higher ROS loads in WAT, with reduced activity of dismutases, peroxidases, and other antioxidants (from Nrf2 inhibition) causing proliferation of lipid peroxidation, decreases in adiponectin, and adipocyte DNA damage in addition to lipogenesis via a putative Acly, Scd1, Fasn, and Acaca-equivalent pathway (Figure 1) [27]. In MUHO, the action of LPA4 within WAT has been shown in mice to promote fat tissue expansion through the Ga12/13-coupled receptor, increasing ROS-related PPAR*γ* activity; thus, MHO phenotypes may be able to suppress lipogenic induction of WAT through reduced ROS-mediated inflammation (Figure 1) [28,29]. Mitochondrial oxidative stress also has a deleterious effect on adipocytes in BAT, which uses a thermogenic effect via UCP1 and generation of batokines and/or other factors (e.g., VEGF, FGF21, etc.) to help maintain basal metabolism and insulin sensitivity (Supplementary Table S1) (Figure 1) [30,31]. However, decreased *β*-adrenergic capacity, BAT lipid droplet formation indicative of oxidization, and reduced adiponectin may result from excess ROS in this tissue [22]. A basic mechanism of ROS-mediated damage in obesity is shown in Figure 1.

It is important to note that, with regard to oxidative stress, while MHO is more metabolically healthy than the insulin-resistant condition of MUHO, it is not a state of total health as the ROS associated with excess body fat cause chronic lipoprotein damage that gives eventual rise to atherosclerosis and risk of strokes or other cardiovascular events [34,35,36]. Additionally, C-reactive protein and other cardiovascular damage markers that result from ROS are often elevated compared to normal weight individuals [37]. Furthermore, higher levels of circulating blood lipids and uric acid (hyperuricemia) in the obese, regardless of metabolic status, facilitate damage to both lipids (e.g., nitrosylated lipoproteins) and vascular endothelium [38]. However, in the short-term, MHO carries comparatively lower risks of ROS generation and these patients can more easily undergo exercise and diet interventions to further reduce oxidative stress generated by obesity.

In East Asia: While browning of WAT into a calorie-metabolizing, “beige,” BAT-like phenotype can occur through the mineralocorticoid receptor, ERK1/2, MAPK, and AKT, the long-term implications of this shift are not clear and the effect of beige fat in East Asian phenotypes with regard to MHO has not been detailed in sufficient studies (Supplementary Table S1) [22]. However, studies in China, Japan, and South Korea have found solid links between ROS and obesogenesis, even in children, that may result in eventual MUHO pathogenesis [39,40,41]. Studies in Asians with regard to blood-based biomarkers of cardiovascular disease (e.g., damaged lipoproteins, endothelial death factors, C-reactive protein, etc.) will be useful in detailing the effect of ROS on the Asian phenotype.

### 2.4. Environmental Pollution

Associations of air and water pollution with obesity may stem from immune-mediated reactions to particles in the air (asthma, COPD) or interactions between chemicals and gut flora as has been observed in animal studies [42,43]. A 2016 systematic review of 35 human cohort studies found that 46% reported positive links between environmental pollution and obesity, similar to a study in 98 people with obesity and 47 normal weight volunteers which found at least some associations between liquid organic pollutants (i.e., pesticides/herbicides) and obesity-relevant biomarkers, such as insulin resistance [44,45]. Meanwhile, a 2014 systematic review of 19 human studies did not find concrete associations between polychlorinated biphenyls, dichlorodiphenyltrichloroethane, and obesity [46]. However, bisphenol A (BPA), a commonly used chemical in the manufacture of polycarbonate plastics, has been found in multiple studies to increase adiposity, with one representative Canadian study of 4733 adults finding an odds ratio of 1.54 [95% CI: 1.002–2.37] with regard to urinary BPA and BMI-defined obesity [47].

In East Asia: The BPA-obesity link was mirrored in a Korean study of 10,021 volunteers in which obese adults had significantly higher BPA levels in their urine and another Korean study of 3782 adults that found additional correlations between paraben levels and type 2 diabetes/obesity risks [48,49]. Clearly, ground/water-borne pollutants, preservatives, and plasticizers may bioaccumulate in lipid tissue, but parabens have been found to concentrate in fingernails, especially those of women, and the sequestration of such products may have an endocrine or immune-disrupting effect that has yet to be fully determined in Asians, especially with regard to the MHO to MUHO shift [50].

Air particulate matter (pm), as defined by size in microns (e.g., pm2.5, pm10), may have a more solid connection to obesity through multiple factors. First, air pollution prevents outdoor exercise, especially in asthmatics or those with allergies (who are already at increased risk of obesity). Second, ROS generated by infiltration of fine particulate matter into the body could promote cumulative damage to the mitochondria and, by extension, the overall energy metabolism [51]. Finally, damage to the cardiopulmonary system by pm2.5 could reduce exercise capacity crucial in maintaining MHO status by increased fibrotic damage [52].

In East Asia: A study using Chinese data from 13,741 adults over a 26-year-period determined that every microgram of pm2.5 increase resulted in a 0.27% rise in BMI via a lack of outdoor exercise [53]. Another Chinese study of 91,121 adults similarly found that each 10-microgram increment of pm2.5 pollution resulted in an 8% increase to the obesity risk [54]. This was mirrored in an Asian study that found, over 9 years, significant associations between airborne sulfur dioxide levels, high blood pressure, and type 2 diabetes [55]. Additionally, high pollution levels in Asia that affect the amount of available sunshine may also increase obesity, as seen in a 2022 study of 47,204 Chinese adults in which the association between pm2.5 and obesity was dependent on available sunshine duration [56].

### 2.5. Seed Oils and Allergies

Traditional use of unrefined animal fats (e.g., butter or lard) has given way over the past 70 years to the use of highly refined, easily oxidized seed oils (rapeseed, safflower, sunflower, and soybean) chosen for their high smoke point and neutral taste. However, animal studies have reported that linoleic acid in refined soy oil causes both fatty liver and obesity while black seed oil (cold-pressed and unrefined) has been shown to reduce this effect via upregulation of antioxidant master transcription factor Nrf2 [57,58,59]. Unrefined oils (especially fish oil) high in polyunsaturated omega 3 long-chain fatty acids (e.g., EPA/DHA), inversely to seed-based cooking oils, have been repeatedly shown to reduce cardiovascular disease (CVD) risk, chronic inflammation, and obesity risk in animals and humans alike [60]. Cumulative intake of oxidized oils may thus increase metabolic damage and spur progression of MHO to MUHO in populations that rely on such high-heat methods of cooking.

In East Asia: High-heat seed oils may not be causative for all Asian obesity trends since only the Chinese cooking styles features foods fried in excessive oil at high temperatures, which could introduce ROS or other oxidized oil byproducts into the body [61]. A study of 15,022 Chinese adults over 14 years found positive associations between refined lard, peanut oil, canola oil, and sesame oils and type 2 diabetes, indicative of metabolic damage while, conversely, Korean traditional cooking features addition of oil after preparation and would not be a source of ROS from oxidized oils [62,63]. Japanese traditional cooking similarly relies on low oil use but recent increases in animal product consumption and the popularity of Western/Chinese cuisine featuring heavy seed oil use in both Japan and South Korea may be of concern in preventing MUHO pathogenesis.

Obesity, often associated with allergies (even in children), mediates allergic pathogenesis due to activation of chronic inflammatory mechanisms and generation of ROS via sustained hyperglycemia. Another putative mechanism elucidated in animal studies is the breakdown in the intestinal barrier system via the PPAR*γ*/NF-*κ*B pathway and also a higher circulating IgE level [64,65]. The sustained inflammation caused by repeated allergen challenge and immune dysregulation may, thus, synergistically increase the risks associated with obesity under conditions of hyperglycemia and subsequent ROS generation.

In East Asia: A study of 1772 Japanese children found that girls with overweight status were more likely to self-report food allergies and a Chinese study of 3327 children in Wuhan, China found a link between obesity and allergic rhinitis [66,67]. A Korean mental health study of 703,869 children found that, of 440,411 enrolled participants with some form of allergic disease (atopic dermatitis, rhinitis, or asthma), 21,836 (~5.0%) had comorbid obesity [68]. Specific links between MHO, the East Asian phenotype, and allergies are scarce in the literature.

### 2.6. Hormonal Changes, Age, and Menopause

**Figure 2:**
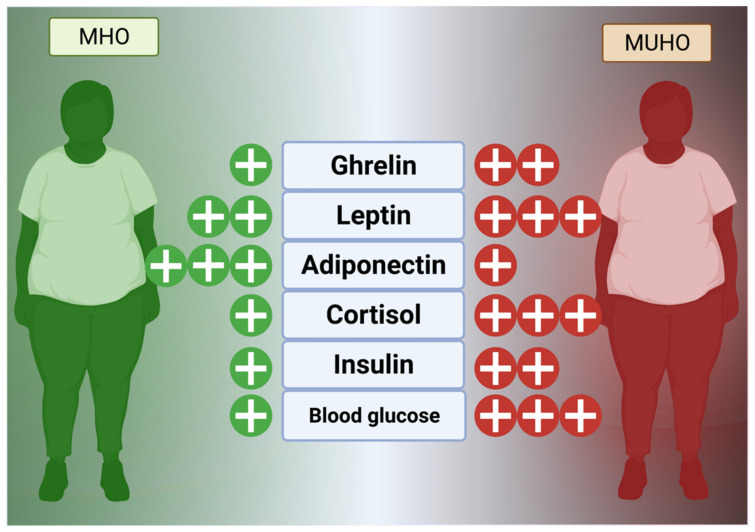
**Hormonal Effects in MHO vs. MUHO**. A delicate biochemical homeostasis between leptin, ghrelin, and adiponectin controls energy intake in addition to the action of insulin. Resistance to leptin signaling increases hunger by upregulating ghrelin while adiponectin may be increased in MHO vs. MUHO [75]. Created in BioRender.com.

Hormones, as a comparatively slower but wide-reaching chemical messenger system, play a key role in obesogenesis and maintenance of excessive fat stores. In general, the hunger and fat management mechanism is currently known to consist of extensively reviewed hormones, such as glucagon-like peptide 1 (GLP-1), visfatin, ghrelin, cholecystokinin (CCK), leptin, and enterostatin, that reside in the digestive tract and fat to control satiety and peristalsis in concert with the hypothalamus via vagus nerve signaling (Figure 2) [69,70]. Additional effects are mediated by cortisol, a circadian hormone released in excess under times of stress that mediates insulin sensitivity and feeding behavior [71]. Cortisol, as a link to the hypothalamus-pituitary-adrenal axis, can be permanently affected by childhood or adult stress (social, occupational, emotional, etc.) and maintain dysregulation, stimulating ghrelin, inflammation, and appetite even if it normally modulates inflammation in a dual-phase, non-linear fashion [72,73,74].

The neurohormonal, homeostatic leptin mechanism is known to be dysregulated in obese individuals, with leptin resistance being a major cause of excessive calorie consumption. Synthesized within fat tissue, leptin functions to signal the hypothalamus that fat storage has occurred, and, in this manner, signals satiety to the ventromedial nucleus and arcuate nucleus centers [75,76]. Ghrelin, conversely, signals the need for feeding behavior and regulates bodily functions, such as sleep-wake circadian rhythm [70,76]. Adiponectin, a related adipokine (protein hormone) released from WAT, modulates insulin sensitivity through AdipoR1 and AdipoR2 receptors on diverse cell types and was found important for blunting the transition from to MUHO from MHO in studies of 822 US adults as well as 1137 children with obesity in China (Figure 2) [77,78,79]. It has also, controversially, been reported to modify appetite, either increasing or decreasing depending on the study, but this effect may be due to localization (serum vs. WAT vs. liver) and other key factors (such as current glucose in the brain) or the presence of other lipid-based factors [80] (Figure 2).

Theoretically, increased leptin from obesity should cross the blood-brain barrier and suppress appetite at the neurochemical level but obese individuals are almost always leptin resistant much in the same manner that diabetics are insulin resistant [81]. However, people with MHO are more likely to have lower leptin levels than those with MUHO [79]. Although various associations have been reported, including epigenetic regulation of leptin production by C-reactive protein, modifications to the blood–brain barrier may play a role and leptin receptor regulation in the hypothalamus may also be causative [81,82]. Animal studies have shown that leptin analogs, co-administered with amylin, CCK, and other pro-anorexic factors may be more efficacious in the severely obese than leptin alone, since weight loss does not rescue the effect of leptin on CCK1 receptors and amylin may be an important backup mechanism [83,84,85]. In humans, studies have shown that MUHO sufferers have higher leptin resistance than MHO sufferers, supporting the idea that leptin is a key part of the obesogenic progression to unhealthy obesity [86]. High adiponectin, as a chief insulin sensitizer, has been shown in several human studies to be associated with the MHO phenotype compared to MUHO [87,88]. Clearly, the implications of the leptin/ghrelin/adiponectin triad are of great importance in delineating the shift from normal weight into MHO and then to MUHO. Future studies of exogenous regulators of these hormones will be useful for clinical development of long-term weight loss drugs that have fewer side effects.

Sex hormone changes, especially in the overweight/obese, may also be a synergistic factor in recalcitrant obesity. This is due to the regulation of fat deposition by estrogen away from the viscera and into gluteal storage areas where impact on systemic inflammation can be lessened [89]. As estrogen deficiency is a noted and well-studied risk factor for metabolic syndrome/unhealthy obesity, its ability to suppress inflammation could also be a key factor in the low metabolic risk of premenopausal women compared to men [90].

In East Asia: Increases in perceived stress from the industrialization of East Asia, work, finances, and the fragmentation of families due to employment migration might exacerbate levels of obesity-related stress hormones. As numerous studies have reported similar results with regard to Asians, BMI, and leptin resistance, further exploration of resistance pathogenesis might give new insights on therapies to prevent the MHO to MUHO shift in East Asia.

Sex hormone studies may be a key component of East Asian MUHO pathogenesis as an Asian review of population-based studies demonstrated that low testosterone and obesity are linked, a conclusion supported by a study of 4081 Chinese adult males, which found that low testosterone levels translated to a three times higher risk for obesity and individuals with MUHO had the lowest testosterone [91,92]. For women, the loss of protective estrogen may both precipitate obesity and drive the MHO to MUHO shift as seen in a study of 646 Chinese postmenopausal women, 48% of whom had abdominal obesity [93]. This study also found that high stress, a lack of previous breastfeeding experience, and poor sleep were also associated with obesity [93]. Additionally, a Korean study of 929 peri- or post-menopausal women found that the most severe symptoms were in obese patients, supporting the link between estrogen loss and obesity [94]. However, a study in 345 Korean postmenopausal women found that estrogen replacement therapy had no links with lower cholesterol or lipid levels in overweight women [95]. Meanwhile, MUHO associated with polycystic ovary syndrome (PCOS), an autoimmune disorder found in women that features pan-androgen hormonal dysregulation, was found in a study of 94 women to be reliant on higher testosterone levels while those with MHO and PCOS had lower androgens [96]. However, in Asian studies, while a Chinese study of 719 PCOS sufferers found one-third to be insulin resistant, these volunteers were not classified according to MHO criteria (thus links to androgens, PCOS, and MHO could not be established) [97]. Future studies in Asian women regarding the effect of PCOS phenotypes on MHO may be useful to examine the role of estrogen and androgens in this transition.

### 2.7. Immune Factors

The immune system is a complex population of organs, biochemistry, and interplay between specialized effector and somatic cells. Obesity, as a chronic inflammatory condition, has been well reported to maintain increased systemic interleukin (IL)-2, IL-4, IL-6, TNF*α*, IFN*γ*, and GM-CSF levels in a manner modulated by physical activity (Supplementary Table S1) [98]. These cytokines are secreted by effector cells (e.g., polarized M1-type macrophages), as well as lipid cells, and activation of fatty acid-sensitive Toll-like receptors (TLR), such as TLR-2 and TLR-4, are reported to play a central role in obesogenic, chronic immune dysregulation [99,100]. Interplay between hormones and cytokines may also be important as TNF*α*, which reduces insulin sensitivity and has been shown to be overexpressed in people with obesity, is partially mediated by leptin in addition to IL-2, and IL-6 cytokines that increase T-cell expansion and liver disorders [101,102,103]. Additionally, microRNAs generated by adipose tissue, such as miR155, are instrumental in activating the inflammatory response while extracellular matrix proteins, especially MMP-9, play crucial roles in the collagen deposition that facilitates fat storage [100,104]. However, therapies which reduce immune-mediated inflammation, such as anti-TNF*α* antibodies or corticosteroids (injected, oral, inhaled, etc.), have been paradoxically shown to increase inflammation and fat mass, an effect that persists even when locally administered [105,106]. Thus, the complex interactions between diverse immune receptors (e.g, TLR), other receptors (like the glucocorticoid or mineralcorticoid receptors), and microRNA could function as integral backup signals for obesogenesis independent of homeostatic energy intake control.

In a meta-study examining 15 reports of 5421 total cases of type 2 diabetes, IL-6 was strongly related, a finding supported by a subsequent multi-ethnic analysis of 260,614 diabetics which found that Asp358Ala mutations in the IL-6 receptor (IL-6R) were correlated with type 2 diabetes [107]. Coupled with a meta-study of 11 reports of associations between cytokines (such as IL-6), periodontitis, and obesity, a picture of cytokine dysregulation and inflammation in the obese, regardless of co-morbidities, emerges [108]. In MHO, on the other hand, levels of inflammatory cytokines have been reported to be lower than in MUHO and a microRNA-regulated pro-inflammatory state has been shown in murine models to be conducive to an MHO-to-MUHO phenotypic switch, especially with regard to miR155 [104,109]. Additionally, the MHO phenotype maintains WAT vascularization comparable to normal weight phenotype and capillary density is also maintained, leading to sustained cardiovascular capacity [100]. This, however, does facilitate adipogenesis even if the growth factors released help sustain suppression of inflammation [100]. While anti-inflammatory antibody therapies that paradoxically increase inflammation may play a role in shifting MHO to MUHO, blocking adipose-derived microRNAs (e.g., miR27a) responsible for immune dysregulation and chronic inflammation may be attractive for therapies that could prevent the shift to unhealthy obesity [110].

In East Asia: Asian studies, such as a study of 217 adults, found elevated IL-33 (Th2, pro-allergic factor) in obese patients while a study linked IL-6 polymorphisms, obesity, and osteoporosis risk in elderly Chinese women [111,112]. The effect of immune dysfunction in lipid tissue on obesogenesis in children has been extensively reviewed by Asian authors [113].

### 2.8. The Microbiome

**Figure 3:**
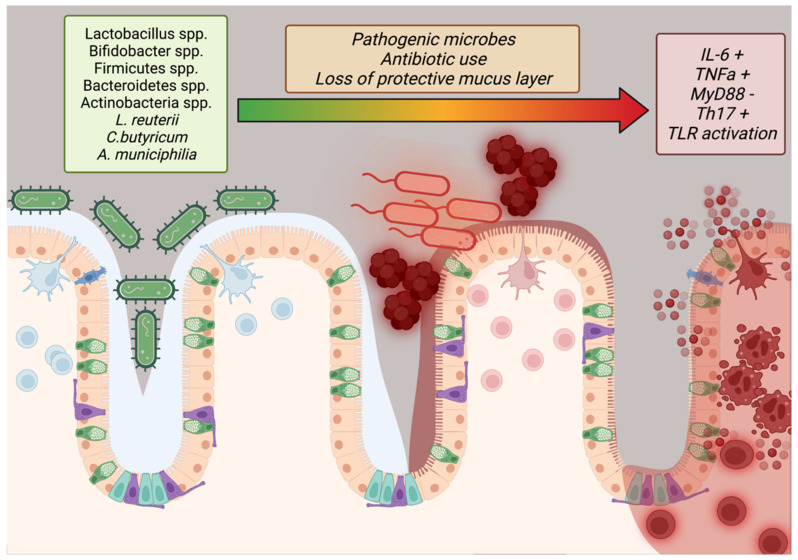
**Importance of barrier function in preventing obesity.** Diverse symbiotic species of bacteria protect the digestive tract by crowding out pathogens as well as maintaining the mucus barrier and promoting immune tolerance by dendritic sampling. Pathogens, antibiotics, and loss of the mucus layer degrades the integrity of the epithelial barrier via lowered MyD88 and immune recruitment creates sustained inflammation that may promote obesity [115,117]. Created in BioRender.com.

Only within the last 40 years has the importance of the microbiome to human health conditions (such as obesity) been intensely studied. Studies have shown that imbalances in the gut flora, especially with regard to high Firmicutes to Bacteroidetes ratios, are indicative of obesity but the complete effect of stable microbes in the human digestive system have not been fully elucidated [114,115]. Additional studies have reported that microbial maintenance of the mucous barrier within the intestines, bolstered by mucinogens such as *Akkermansia muciniphilia*, reduces systemic inflammation by reducing allergen ingress from digesting food while *Lactobacillus reuterii*, *Clostridium butyricum*, and other potentially transient species may contribute to barrier function and lipid/glucose homeostasis by regulation of IL-10, GLP1, GLUT2, and TLR2/AMPK pathways (Suppplementary Table S1) (Figure 3) [115,116]. In addition, the epithelium of the small intestine has tight junctions to prevent ingress of particles into the bloodstream, a function mediated by MyD88 secreted by Paneth cells (Figure 3) [117]. This is in addition to the normal mediation of immune tolerance via the “host-gut microbial cross-talk” mechanism [118]. Disruptions in this delicate balance (dysbiosis) or encroachment by pathogenic Staphylococcus, Shigella, or other species may remove the protective layer of mucus and disrupt tight junction integrity, causing immune recruitment that further damages the epithelium and exposes the body to allergens that could sustain obesogenic inflammation (Figure 3) [117]. To this end, researchers have progressed beyond promising animal studies to conduct clinical trials on patients with obesity with supplements featuring these key bacteria. A random clinical trial in 120 people with Lactobacillus and Bifidobacterium spp.-containing Lab4P supplements for 6 months found greater weight loss, lower blood pressure, and lower LDL-C versus controls while a proof-of-concept study in 32 obese volunteers showed significant improvements in insulin sensitivity [119]. A systematic review of 14 reports featuring various Lactobacillus strains in weight loss trials found that 9/14 of the trials showed significant weight loss and that these effects were strain and diet dependent [120].

In East Asia: In Japan, a study of 100 adult men with obesity and prediabetes found that *L. casei* strain Shirota-fermented milk administration resulted in glycoalbumin and HbA1c reductions after 8 weeks even though blood sugar reductions were not seen [121]. Other trials in East Asia featuring probiotic interventions for MHO maintenance are scarce in the literature.

### 2.9. Social Aspect

Bullying and social pressure during childhood have been associated with lifelong obesity in multiple reports. Chronic psychosocial stress, especially bullying that causes hopelessness, estrangement, and anxiety, has been repeatedly shown to engender compensatory binge eating as well as increased pro-inflammatory cytokine environments (e.g., TNF*α*, IL-6, IL-1*β*) that can serve as a foundation for rapid and persistent fat gain [122,123]. A study of 4781 11–15-year-old children in Denmark found that boys and girls with overweight/obese statuses were subjected to about twice the risk of bullying, an effect pronounced among girls with obesity (OR 2.74) [124].

In East Asia: Additional psychosocial stress has also been associated with lower income levels, sedentary lifestyle (excessive TV or screen viewing), excessive study time, and bullying issues in Japan and China [125,126]. A global health survey of 41 low-to-middle-income countries (including SE Asia) found that girls and boys with overweight/obese statuses were 3.4× and 2.48×, respectively, more likely to be bullied [127]. The higher population densities in some Asian cities, coupled with cultural expectations of emotional subjugation, may contribute to higher social stress that could exacerbate obesity or create chronic stress leading to metabolic syndrome in the already overweight [128].

### 2.10. Vitamin and Micronutrient Deficiencies

A lack of vitamins or other micronutrients has been implicated in obesogenesis and animal studies have shown that obesity features a lack of Vitamins A and D, with Vitamin A regulating metabolism through RBP3 and ALDH1A1 and Vitamin D playing key roles in adipokine synthesis, calcium balance, and glucose metabolism [129,130,131]. Vitamin D, shown to be sequestered in fat tissue and, therefore, of low bioavailability in the obese, additionally regulates the immune system through the Vitamin D receptor on T cells, reducing the effect of chronic inflammation on obesogenesis [131,132]. Vitamin B, although involved in energy utilization, has been implicated as a controversial obesogen. Zhou and colleagues asserted in 2014 that fortification of foods with synthetic B vitamins was correlated with increases in weight gain that could be distinguished at a national level, a finding correlated by a study in Iran among 5606 children in 2019 that found increased weight gain with Vitamin B1, Vitamin B3, Vitamin B5, and Vitamin B6, but not folic acid, Vitamin B12, or Vitamin B7 [133,134]. However, conflicting studies have shown that people with obesity often suffer from Vitamin B12 deficiency (in a study of 9075 people) and animal studies have shown the usefulness of B vitamins for weight loss and prevention of hyperhomocysteinemia that could exacerbate any CVD effects of obesity [135,136,137].

In East Asia: With regard to MHO and vitamins, a study of 16,190 Korean adults found that MHO sufferers had lower AST/ALT liver enzyme levels and that, while Vitamin D deficiency was related to insulin resistance, there were no significant differences between MHO and MUHO with regard to Vitamin D levels [138]. In spite of possible antioxidant and immune-regulating benefits, studies have not shown a clear link between vitamin deficiencies and the MHO to MUHO phenotypic shift, especially in Asian populations.

### 2.11. Weight Fluctuation and Rebound Effect

Regardless of the pathogenesis, weight management is the first line of defense against obesity; however, the sustained inability of diet/exercise to reduce body fat over a significant period of time traps many individuals with obesity in a constant cycle of “yo-yo” dieting. This results in homeostatic weight “thermostat” increases after each diet cycle and active resistance to persistent weight loss by reducing energy expenditure and increasing appetite (rebound effect). A meta-analysis consisting of 29 included, long-term weight-loss studies found that half of lost weight is regained after only 2 years while, at the 5-year mark, 80% was found to be regained [139]. Thus, MHO patients placed in a typical diet/exercise program more appropriate for MUHO may actually be harmed by frequent weight cycling as it has been shown in studies to carry an increased risk of both all-cause mortality and cardiovascular events [140].

In East Asia: Although studies on rebound are scarce, several small studies in Japan and China implicated a return to previous overfeeding patterns due to social contact or post-surgical pain that limited activity [141,142]. A large Korean study of 3678 adults, among whom half experienced high weight variability, found increased risk of death and rebound was attributed to homeostatic feedback after weight loss that creates a biochemical milieu favorable to weight gain [140].

### 2.12. Genetic Factors

Studies have discovered multiple genes associated with obesity, even in children. Inherited obesity and polygenic obesity (stemming from multiple and synergistic polymorphisms) are thought to involve brain, signaling, and nervous system genes such as NTRK2, KCNQ1, MRAP2, and ADCY3 that regulate satiety and thermo-caloric energy balance (Supplementary Table S1) [143]. However, single nucleotide polymorphisms in other energy balance genes such as UCP1 (rs1800592, rs10011540) and NPC1 (rs1805081, rs1805082) have also been reported [144]. If such genes are inherited, this could predispose infants to a lifetime of energy dysregulation, hyperglycemia, excessive ROS, chronic inflammation, and risk of both obesity and rapid progression to MUHO.

In East Asia: A study of 1213 Chinese children found that KCNQ1-rs2237897 is associated with cardiovascular and KCNQ1-rs2237892 is associated with insulin resistance risk in MHO while another Chinese study of 1790 MHO children found FTO-rs9939609 or CYP17A1-rs11191548 predictive of cardiovascular risk in addition to GNPDA2-rs10938397 or KCTD15-rs29941 being predictive for insulin resistance [145,146]. A separate Chinese study did find that adiponectin-related gene polymorphisms, especially rs6773957, were related to diet and MUHO, in line with a similar Russian study of 503 obese patients that found similar connections to polymorphisms such as G45G [147,148]. A genome-wide association study of 49,915 Koreans found that LPL, APOA5, CETP, GCKR, CDKAL1, and CDKN2B (all lipid metabolism genes; Supplementary Table S1) were associated with MHO, thus lending weight to the concept that the MHO condition is a discrete genetic phenotype and that shifts to MUHO may involve epigenetic regulation from synergistic internal (reactive oxygen species, stress, hormones, etc.) and/or external (pollution, diet, etc.) sources [149]. Exclusive of other influences, obesity (and a predisposition to some intermediate period of MHO) is certainly heritable, either through alterations in hormones, lipid metabolism, or neurobehavioral aspects, and children of persons suffering from obesity have been found in both Western and Asian studies to be much more likely to suffer from excessive fat storage and obesogenesis than children with normal-weight parents [150,151,152,153,154].

## 3. Conclusions

Our review must acknowledge limitations in that its narrative structure prevented meta-analysis for each listed factor to determine the most important with regard to the shift from MHO to MUHO. Moreover, the Asian phenotype itself is not completely homogenous and, thus, recommendations associated with listed factors are to be taken generally; studies in Asian populations with regard to MHO will need adjust for specific subgroups to fully outline the exact causative factors for each group (e.g., the Iban people of Borneo versus Han Chinese).

Future research into methods to treat obesity in Asia will need to take into account the potential effectiveness of bariatric surgery and the recent development of new, hormone-modulating drugs being developed to counter leptin resistance and ghrelin imbalances [155]. As Asians may suffer disproportionately from metabolic diseases due to lower insulin sensitivity (which triggers weight gain), the use of such drugs after testing in diverse Asian populations may relieve both metabolic and musculoskeletal stress due to weight loading on the hips and legs [4,156]. Of particular importance will be epigenetic and genome-wide association studies to catalog the impact of heritable factors on pediatric obesogenesis, especially in the Asian phenotype

Factors relevant to the Asian population and MUHO/MHO are summarized in Table S1. As obesity rates continue to rise in East Asia due to environmental, food, social, genetic, and other factors, the proportion of Asians with MHO is also expected to rise. However, understanding and intervening in public health areas (nutrition education, pollution control, inflammation control, etc.) may allow for health authorities to prevent the MHO to MUHO shift and reduce MUHO pathogenesis in their respective populations.

## Data Availability

Not applicable.

## Supplementary Materials

The following supporting information can be downloaded at: https://www.mdpi.com/article/10.3390/medicina58091271/s1. Reference [157] is cited in the supplementary materials.
