# Supplementary material for: Factors of Obesity and Metabolically Healthy Obesity in Asia
Source: Medicina (Kaunas). 2022 Sep 13;58(9):1271. doi: 10.3390/medicina58091271 (PMC9500686; doi:10.3390/medicina58091271)
Supplement: Supplementary file 1 [file medicina-58-01271-s001.zip › medicina-1857091-supplementary.pdf]

Table S1. Gene/Protein Abbreviation Table [1].

Gene/Protein	Name
Acaca	Acetyl-CoA Carboxylase 1
Acly	ATP Citrate Lyase
ADCY3	Adenylate Cyclase 3
Adipo R1/R2	Adiponectin Receptors 1& 2
AKT	AKT Serine/Threonine Kinase
ALDH1A1	Aldehyde Dehydrogenase Family Member A1
ALT	Alanine Aminotransferase
AMPK	AMP-activated Protein Kinase
APOA5	Apolipoprotein A5
AST	Aspartate aminotransferase
CCK	Cholecystokinin
CDKAC1	CDK1/Cyclin-Dependent Kinase 1
CDKN2B	Cyclin-Dependent Kinase Inhibitor 2B
CETP	Cholesteryl Ester Transfer Protein
CYP17A1	Cytocrhome P450 Family 17 Subfamily A Member 1
ERK1/2	Extracellular-Related Kinase 1 and 2
Fasn	Fatty Acid Synthase
FGF21	Fibroblast Growth Factor 21
Ga12/13r	G Protein 12 or 13 Receptor
GCKR	Glucokinase Regulator
GLP-1	Glucagon-Like Peptide
GLUT2	Glucose Transporter 2
GM-CSF	Granulocyte-Macrophage Colony Stimulating Factor
GNPDA2	Glucosamine-6-Phosphate Deaminase 2
HbA1c	Hemoglobin A1c
IFNv	Interferon gamma
IgE	Immunoglobulin E
IRS-1	Insulin Receptor Substrate 1
IRS-2	Insulin Receptor Substrate 2
JNK	c-JUN-N-Terminal Kinase
KCTD15	Potassium Channel Tetramerization Domain Containing 15
Keap1	Kelch-Like ECH-Associated Protein 1
KCNQ1	Postassium Voltage-Gated Channel Subfamily Q Member 1
LPA4	Lysophosphatidic Acid Receptor 4
LPL	Lipoprotein Lipase
MMP-9	Matrix Metallopeptidase 9
MRAP2	Melanocortin 2 Receptor Accessory Protein 2
MyD88	Myeloid Differentiation Primary Response 88
NADH	Nicotinamide Adenine Dinucleotide
NADPH	Nicotinamide Adenine Dinucleotide Phosphate
NF-kB	Nuclear Factor Kappa B
NPC1	NPC Intracellular Cholesterol Transporter 1
Nrf2	Nuclear Factor Erythroid 2-Related Factor 2
NTRK2	Neurothrophic Receptor Tyrosine Kinase 2
p38MAPK	p38 Mitogen-Activated Protein Kinase
p65	Transcription Factor p65

PPARv	Peroxisome Proliferator-Activated Receptor Gamma
RBP	Retinol-Binding Protein
Scd1	Stearoyl-CoA Desaturase 1
TLR-2	Toll-Like Receptor 2
TLR-4	Toll-Like Receptor 4
TNFa	Tumor Necrosis Factor Alpha
UCP1	Uncoupling Protein 1
VEGF	Vascular Endothelial Growth Factor

1. Safran, M.; Rosen, N.; Twik, M.; BarShir, R.; Stein, T.I.; Dahary, D.; Fishilevich, S.; Lancet, D. Practical Guide to Life Science Databases. In *The GeneCards Suite*; Springer Nature Singapore: Singapore, 2021; pp. 27–56. https://doi.org/10.1007/978-981-16-5812-9_2.
